# Evaluation of predictions in the CASP10 model refinement category

**DOI:** 10.1002/prot.24377

**Published:** 2014-07-31

**Authors:** Timothy Nugent, Domenico Cozzetto, David T Jones

**Affiliations:** Department of Computer Science Bioinformatics Group, University College LondonLondon, WC1E 6BT, United Kingdom

**Keywords:** protein structure prediction, template-based modeling, protein model refinement, CASP, model quality evaluation

## Abstract

Here we report on the assessment results of the third experiment to evaluate the state of the art in protein model refinement, where participants were invited to improve the accuracy of initial protein models for 27 targets. Using an array of complementary evaluation measures, we find that five groups performed better than the naïve (null) method—a marked improvement over CASP9, although only three were significantly better. The leading groups also demonstrated the ability to consistently improve both backbone and side chain positioning, while other groups reliably enhanced other aspects of protein physicality. The top-ranked group succeeded in improving the backbone conformation in almost 90% of targets, suggesting a strategy that for the first time in CASP refinement is successful in a clear majority of cases. A number of issues remain unsolved: the majority of groups still fail to improve the quality of the starting models; even successful groups are only able to make modest improvements; and no prediction is more similar to the native structure than to the starting model. Successful refinement attempts also often go unrecognized, as suggested by the relatively larger improvements when predictions not submitted as model 1 are also considered. Proteins 2014; 82(Suppl 2):98–111.

## INTRODUCTION

The scope and accuracy of comparative modeling have been increasing steadily over time, but generating predictions that are consistently closer to the native structure than they are to the original templates still proves very challenging. Despite significant improvements in alignment accuracy and more recently in the modeling of large insertions, progress in the so-called “end game” in prediction—adapting purely template-based models to accurately represent the observed time averaged native structure—has been slow. Protein refinement methods developed since CASP9 have focused on a broad range of strategies including molecular dynamics (MD), fragment-based approaches, knowledge-based approaches, elastic network models, and hydrogen bond network optimization.^1^–^16^ Perhaps the most promising advances have come in the design of novel parallel supercomputer architectures comprising substantial numbers of application-specific integrated circuits, which now allow atomistic MD simulations to run for as long as 100 μs.^17^,^18^ In combination with increasingly accurate physics-based force fields such as CHARMM, which have been used to successfully fold structurally diverse sets of fast-folding proteins,^19^–^22^ the major problems limiting the application of MD to protein refinement can now be mitigated to some extent. In view of the recent excitement that these early results have generated, it was disappointing that these methods were not tested in CASP10. Nevertheless, it is worth noting that distributed computing projects such as Folding@home have also achieved similar aggregate ensemble simulation timescales,^23^ while collaborative multiplayer online games such as Foldit have demonstrated that the integration of human visual problem-solving and strategy development capabilities are a powerful approach to tackle computationally-limited problems.^24^

Other than technological development, another problem in the field has been a lack of robust and large-scale benchmarking—which clearly mirrors the issues faced in the early days of CASP. We can only hope that focussing community attention on the endgame problem will have the same level of long term benefit as seen in the template-based modeling (TBM) areas. However, even simple logic tells us that the endgame problem is going to be at least several orders of magnitude harder than the TBM problem, so we may need to be patient.

In an attempt to address the lack of progress in protein model refinement, CASP8 saw the launch of a separate assessment category to identify effective methods and to track advances. For the third time, therefore, the CASP experiment has included a refinement category where the best server TBM predictions are subsequently released as refinement test cases. Groups attempt at improving these structures blindly, with occasional hints about problematic regions. This refinement task differs slightly from the standard definition in the Refs.^1^–^17^ in that the starting models have often already been refined by the original servers. Refinement predictors therefore face the significant challenge of trying to add further value to the prediction, beyond the capabilities of the best TBM groups. For a few targets, the same predictors who produced the starting model also attempted refinement, which clearly produces a problem of diminishing returns for these groups.

Here, we describe the assessment results of the predictions entered at the CASP10 structure refinement category. Models for 27 targets were available to analyze this time, which is close to double the number of CASP9 and targets.^18^ Encouragingly, there was also a large increase in the number of registered groups—from 32 to 50—suggesting that the initiative is drawing increasing attention from the community.

In contrast to the free modeling (FM) and TBM categories in CASP, the released models are usually quite close to the native structures, with differences in backbone conformation, side chain packing as well as local geometry. This necessitates the use of numerical evaluation measures that are more sensitive to subtle changes, whilst also capturing all of the different aspects with which we can assess model quality. Because such features can be optimized separately, we scrutinized model quality through different lenses to properly separate individual efforts and the extent of their success. To this end, we combined an array of standard evaluation measures but strongly emphasized improvement in main chain atom coordinates relative to the native structure. This is reasonable, as it is of little benefit to end users, if the stereochemistry is improved only at the expense of greatly reducing the main chain accuracy of the models.

## MATERIALS AND METHODS

### Target selection

One of the major concerns in CASP9 was the low number of refinement targets, which made it difficult to reach statistically sound and general conclusions. For CASP10, assessors and organizers therefore endeavored to expand the benchmark set, and succeeded in the release of 28 starting models. Table1 lists summary data about the 27 targets that were left in the assessment, after the organizers canceled target TR724 during the prediction season.

**Table I:** Summary of CASP10 Refinement Targets and Accuracy of Corresponding Starting Models

Target	Residues	Method	Model Id	GDT-HA	RMSD	GDC-SC	SphGr	MP	FlexE
TR644	141	X-ray	T0644TS113_1	67.73	2.71	43.46	75.18	2.53	2.87
TR655	175	NMR	T0655TS335_1	49.28	4.65	28.53	51.43	3.83	10.65
TR661	185	X-ray	T0661TS330_5	60.67	2.74	37.50	71.35	1.02	2.13
TR662	75	NMR	T0662TS035_4	63.67	2.03	33.64	76.00	2.47	2.08
TR663	152	X-ray	T0663TSXXX_X	49.34	3.37	26.26	76.97	4.05	2.78
TR671	88	X-ray	T0671TS333_5	36.36	7.72	11.58	44.32	3.68	4.44
TR674	132	X-ray	T0674TS486_1	71.40	3.44	44.17	74.24	2.99	2.82
TR679	199	X-ray	T0679TS330_5	51.63	3.95	30.76	52.26	1.15	9.15
TR681	191	X-ray	T0681TS222_3	58.12	2.27	32.74	63.87	2.89	3.28
TR688	185	X-ray	T0688TS330_3	57.70	2.52	42.49	77.30	1.77	4.71
TR689	214	X-ray	T0689TS463_1	71.73	1.66	42.02	87.38	3.18	1.57
TR696	100	X-ray	T0696TS277_4	52.00	3.52	26.31	50.00	3.04	4.07
TR698	119	X-ray	T0698TS108_4	45.38	4.65	25.68	65.55	2.73	5.40
TR699	225	X-ray	T0699TS439_3	65.44	2.21	33.61	77.33	2.77	4.37
TR704	235	X-ray	T0704TS286_1	49.15	2.78	23.25	73.19	3.10	2.26
TR705	96	X-ray	T0705TS476_1	44.79	4.71	22.11	37.50	3.17	20.74
TR708	196	X-ray	T0708TS081_2	72.83	4.63	45.51	82.14	2.65	3.95
TR710	194	X-ray	T0710TS028_1	53.87	2.44	36.28	77.32	0.56	2.60
TR712	186	X-ray	T0712TS333_5	81.45	1.99	55.15	88.17	2.76	3.63
TR720	198	X-ray	T0720TS330_3	41.41	8.52	25.58	46.97	1.33	10.08
TR722	127	X-ray	T0722TS330_1	38.58	4.42	16.14	89.76	0.99	0.97
TR723	131	X-ray	T0723TS439_1	67.37	2.23	37.72	84.73	2.21	2.41
TR738	249	X-ray	T0738TS424_5	74.60	1.40	50.36	93.98	2.38	0.95
TR747	90	X-ray	T0747TS286_4	65.28	1.96	37.96	67.78	2.02	4.69
TR750	182	X-ray	T0750TS124_1	56.73	2.12	34.80	79.67	2.47	1.76
TR752	148	X-ray	T0752TS292_2	76.01	1.50	43.05	79.73	1.52	0.89
TR754	68	NMR	T0754TS035_1	58.09	2.41	19.97	82.35	2.65	2.48

After careful visual inspection, we selected as refinement challenges those TBM targets or their structural domains (1) that were relatively small, with less than ∼250 amino acids and few missing residues; (2) that exhibited limited crystal contact distortions if they were solved by X-ray crystallography; (3) that had a tight conformational ensemble if they were studied by NMR spectroscopy. For proteins meeting such conditions, we sifted the corresponding server models to select a suitable starting model, which passed stereochemistry and quality checks using Molprobity^19^ and ProSA^20^ and which were close—but not too close—to the experimental structure.

This last test was based on both GDT-HA^21^ and FlexE^22^ scores relative to native, because LGA^23^ alignments are based on rigid-body superposition and therefore can only partially take into account protein intrinsic flexibility or energy landscape. In contrast, FlexE is based on an elastic network model and uses the deformation energy as measure of the similarity between two structures. We used FlexE scores to discard model that were deemed within the thermal ensemble of the target structure—that is when the estimated energy difference was ≤0.89 kcal/mol/residue.

Finally, we attempted to vary as much as possible the source of the initial models, as to avoid biases against groups participating in both TBM and refinement categories. Unfortunately, in some cases our choice of initial model was limited to those from a small number of the best performing 3D modeling servers, but we ended up picking at most five predictions from the same group—namely 330 (BAKER-ROSETTASERVER). One starting model (for TR663) was not generated by any of the servers. This model was generated by the assessor's group using additional distance constraints from the actual experimental structure. Our original plan was to extend the range of targets using structural data available to the assessors but not yet released to predictors that is to come up with starting models when none of the servers had produced accurate enough results. In practice, however, it quickly became apparent that we would probably have sufficient starting models selected from real server predictions, and so further “hybrid” models like TR663 were not required. We kept TR663 in the benchmark because there was no evidence that the data for this target were skewed in any way.

### Model quality evaluation measures

The Protein Structure Prediction Center estimated how well submitted 3D models fitted the corresponding target structures, as well as other stereochemical features observed in the PDB.^24^ All structural alignments were calculated with LGA^21^ in sequence-dependent mode, while model stereochemistry was assessed with MolProbity.^19^ The results of such preliminary analyzes can be found at http://predictioncenter.org/download_area/CASP10/SUMMARY_TABLES/TR_all.tar.gz. Similar to the previous round of the experiment, we mainly focused on:

- GDT-HA score, which is the average of the percentage *c*_d_ of predicted Cα atoms within *d* Å from the corresponding experimental positions. The *c*_d_ values result from independent structural alignments between the model and the target structure with distance cut-offs of 0.5, 1, 2, and 4 Å.
- Root mean square deviation (RMSD) between the predicted Cα atom locations and the corresponding ones in the target structure after optimal superposition.
- GDC-SC score,^25^ which is the weighted mean of the percentage *c*_d_ of predicted side chain atoms within *d* Å from the corresponding experimental positions. For each side chain only one atom is used in the calculation and its choice is amino-acid-dependent. In this case, the *c*_d_ values come from 10 different alignments of the target and the model with distance thresholds of 0.5, 1.0, 1.5, … 4.0, 4.5, and 5 Å.
- SphereGrinder (SphGr) score,^26^ which is calculated from local comparisons of predicted and observed atomic coordinate subsets. For each residue in the target structure, all atoms within 6 Å of its Cα in the experimental and modeled structures are aligned and the fraction *f* of predicted atoms within 2 Å of their counterparts is retained. The final score is obtained by averaging such intermediate values.
- MolProbity (MP) score,^19^ which combines the log-scaled counts of all-atom steric clashes, atypical rotamer conformations and unfavored backbone torsion angles in each prediction.

### Group ranking and comparison procedures

For each 3D model *p* in the set *P* of predictions and for each quality measure *Q*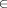 {GDT-HA, RMSD, GDC-SC, SphGr, MP} described above, we first calculated the difference Δ_Q_(*p*) = *Q*(*p*) − *Q*(*s*) relative to the starting model *s*. Then, we converted them into robust *Z*-scores on a target-by-target basis using the formula: 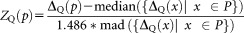
where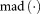 returns the median absolute deviation of its arguments: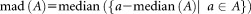. Higher *Z*_Q_(*p*) values indicate more accurate predictions if and only if *Q*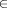 {GDT-HA, GDC-SC, SphGr}, which reflect the different nature of the RMSD and MP score.

Similar to previous assessments in this and other categories, we tried not to discourage groups, which experimented with adventurous refinement strategies and therefore achieved low raw scores. Consequently, we decided to clip the distributions of *Z*_Q_ scores to zero—that is to set to zero any negative *Z*_Q_ for *Q*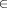 {GDT-HA, GDC-SC, SphGr} and positive *Z*_Q_ for *Q*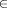 {RMSD, MP}. We finally calculated the overall score: 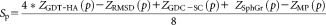
where *Z*_RMSD_ (*p*) and *Z*_MP_ (*p*) are subtracted in line with the above observations.

We performed statistical comparisons of group performance based on individual evaluation measures. Specifically, we tested the null hypothesis that prediction group A does not perform better than B with a one-tailed Wilcoxon signed rank tests on paired Δ_Q_ values at a significance level *α* = 0.01 with no correction for multiple testing. All these statistical analyses were carried out with in-house *R*^27^ scripts.

### Naïve predictions

For each target, we used the starting model itself as a baseline prediction of protein structure refinement. For each evaluation measure, these naïve predictions were assigned target-based *Z*-scores using the statistics that we had separately estimated from the participating group 3D models.

### Molecular replacement

We assessed whether models could be used to solve the phase problem in X-ray crystallography via molecular replacement (MR). MR can make atomic coordinate assignments to a protein of interest by fitting an approximate three-dimensional model of its structure to the observed diffraction pattern. Traditionally, solved homologous proteins are used as search models, but comparative models of the target structure have proved more effective in a number of recent studies. Typically, algorithms based on maximum likelihood and multivariate statistics are used to score the rotation and translation functions, producing the highest correlation when the known and unknown structures are in similar orientations.^28^ We used the log likelihood gain (LLG) score to measure how much better the model explained the data as compared to a random atomic model. Larger values indicate a good fit, with LLG > 60 almost always resulting in a solved structure.^29^ The LLG scores were calculated using the Phaser package from Randy Read's laboratory, and were kindly provided to us by Gabor Bunkoczi via the Prediction Center.

### Hydrogen bonding evaluation

As an additional test of improved model quality, we also compared hydrogen-bonding patterns between submitted predictions and target structures relative to the released starting models. To this end, we first assigned hydrogen bonds (H-bonds) to all experimental and predicted structures with HBPLUS,^30^ and then scored each model based on its ability to reproduce the reference list of hydrogen bonds in the target. This binary classification task was assessed by calculating for each prediction: the number of H-bonds in the model that are also found in the target (TP); the number of H-bonds in the model that are not present in the target (FP); and the number of H-bonds in the target that are missing in the model (FN). From these figures we obtained precision, recall and F-measure respectively as: 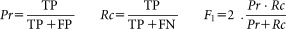


For each 3D model, such statistics were derived considering both all atom pairs and main chain atom pairs only. We then turned the *F*_1_ scores into robust *Z*-scores as described above, clipped the negative values to zero, and finally scored each group by the median of such distributions.

## RESULTS

### Overall results

**Figure 1:**
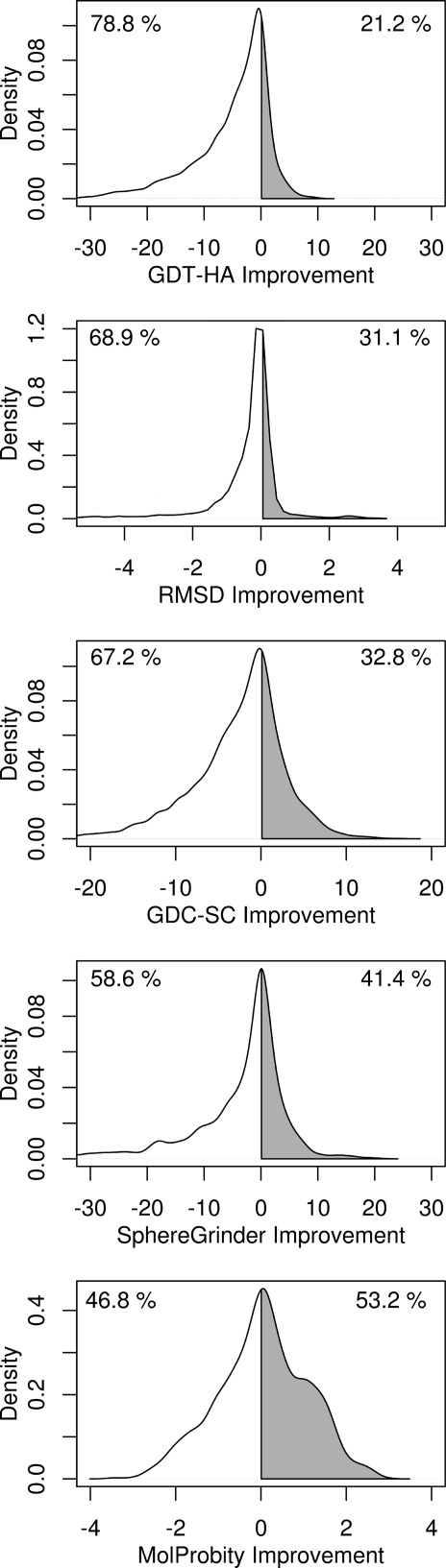
Kernel density plots showing aggregate results by score, from all models submitted by all groups. Numeric values indicate the percentage of time the model was made better or worse than the starting model for each metric.

Figure 1 shows the aggregate results of refinement in CASP10 across all targets submitted by all groups for each of the five assessment metrics. For all metrics apart from the MP score, more models are made worse than are improved, with distributions skewed to the left, indicating there are more big failures than big improvements. These results would suggest that on average, it is not worth refining targets since the average change in four out of five metrics results in a worse model, although clearly this analysis ignores variation between individual groups. Where there is success, scores vary significantly in the frequency with which they were improved, with GDT-HA showing improvement in less than 22% of all predictions, while the MP score was improved in over 53% of predictions, with side chain positioning lying in between. Improving backbone positioning is clearly more challenging than improving physicality and local structure, indicating that future strategies that focus on improving the overall fold stand to benefit the most. There is a concomitant impairment in the magnitude of improvement, with a maximum ΔGDT-HA of only about 10 units. Given that the average GDT-HA across all starting models is less than 60, predictors are currently only making small inroads into overall target refinement.

**Figure 2:**
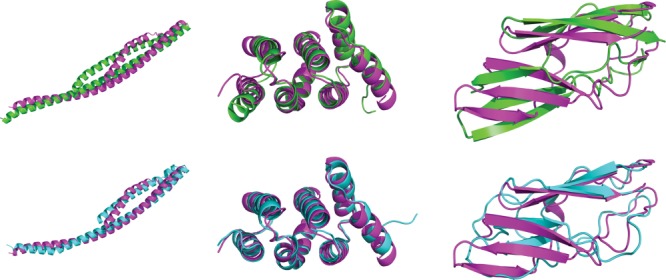
A selection of the best models submitted in CASP10. The native structure is colored magenta, the starting model is colored green (top row) and the model is colored cyan (bottom row). Left: TR722TS149_5, ΔGDT-HA 7.88. Center: TR723TS049_4, ΔGDT-HA 9.16. Right: TR671TS085_3, ΔGDT-HA 9.95. Structures were rendered using the PyMOL Molecular Graphics System.

There are, however, clear examples of successful predictions. Figure 2 shows three models where large increases in GDT-HA were observed, and in most cases all other metrics were also improved as compared to the starting model. Visual inspection clearly reveals that both loop and secondary structure elements have been moved closer to the native state. These examples represent some of the best predictions submitted in this category of CASP10, and although no groups were able to consistently improve starting models to such an extent, the presence of these results is encouraging.

### Model quality evaluation and group ranking

**Figure 3:**
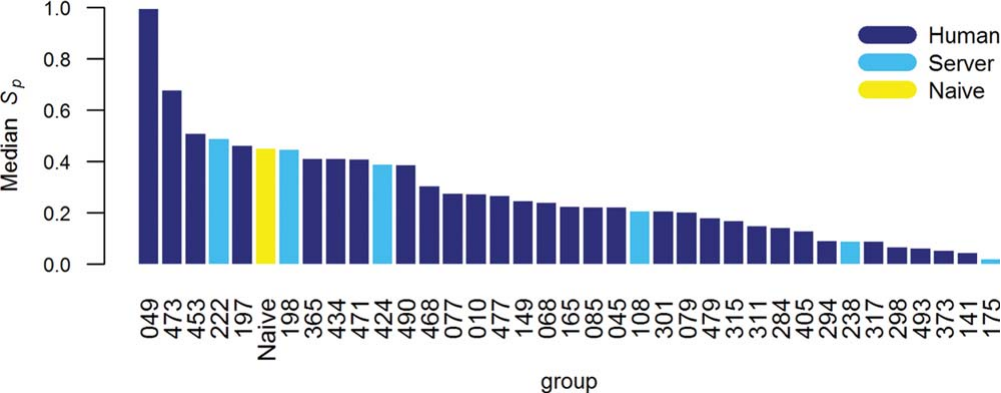
Median *S*_p_ scores for groups participating in the structure refinement category. Bars are shown only for methods that attempted the refinement of 23 targets or more.

Similarly to the approach used by the CASP9 assessor, we evaluated the refinement category predictions through different measures, which examine complementary aspects of model quality including overall fold, as well as the distributions of interatomic contacts and of φ, ϕ, and χ dihedral angles. We discarded from all analyses 22 submissions with fewer atomic coordinates than the corresponding starting models—that is they had missing residues or they were not all-atom models. Therefore, the results discussed below relate to 47 out of the 50 registered groups—all bar 179 (Lenserver), 444 (Lenregular), and 482 (biouv). The models labeled as the most reliable during the prediction season—“Model 1” in the usual CASP jargon—formed the basis for our principal evaluation as detailed in Materials and Methods section. We selected 36 teams that had tried to refine at least 23 targets and show in Figure 3 how they fare in comparison with the naïve approach. The corresponding group names and some high level statistics are reported in Table2; more detailed information for these and statistics for all other assessed predictors are available in the Supplementary Information Table S1. A few groups achieved higher scores than the baseline predictor of model refinement: 049 (FEIG), 473 (Seok), 453 (KnowMin), 222 (MULTICOM-CONSTRUCT), and 197 (Mufold).

**Table II:** Summary Information of the Top Ranked Groups in the CASP10 Refinement Category

Group Id	Group name	Type	Predicted targets	Median *S*_p_ score
049	FEIG	Human	27	0.992
473	Seok	Human	27	0.674
453	KnowMIN	Human	27	0.506
222	MULTICOM-CONSTRUCT	Server	27	0.486
197	Mufold	Human	27	0.460
198	chuo-fams-server	Server	26	0.444
365	chuo-fams	Human	27	0.409
434	chuo-fams-consensus	Human	27	0.409
471	chuo-binding-sites	Human	27	0.407
424	MULTICOM-NOVEL	Server	27	0.388
490	Zhang_Refinement	Human	27	0.384
468	Mufold-R	Human	27	0.303
077	FLOUDAS	Human	27	0.274
010	TSlab-refine	Human	27	0.271
477	BAKER	Human	27	0.264
149	wfFUIK	Human	23	0.245
068	FOLDIT	Human	23	0.237
165	Void_Crushers	Human	23	0.223
085	Anthropic_Dreams	Human	23	0.221
045	Zhang_Ab_Initio	Human	27	0.220
108	PMS	Server	27	0.205
301	LEE	Human	27	0.205
079	TASSER	Human	27	0.201
479	Boniecki_LoCoGRef	Human	26	0.179
315	keasar	Human	24	0.168
311	Laufer	Human	27	0.147
284	Schroderlab	Human	26	0.140
405	Mufold2	Human	27	0.127
294	chuo-repack	Human	27	0.091
238	chuo-repack-server	Server	26	0.088
317	SHORTLE	Human	27	0.087
298	MidwayFolding	Human	23	0.066
493	LEEMO	Human	26	0.061
373	Kim_Kihara	Human	27	0.052
141	Bates_BMM	Human	27	0.042
175	FRESS_server	Server	27	0.020

We then investigated the stability of the final ranking under a wide range of conditions, which may reflect different assessment options—see Supporting Information Table S2. Although the conversion of raw scores—the Δ_Q_ values in our case—into target-based *Z*-scores is well-established practice at CASP,^31^ the assessors made use of parametric statistics in CASP8 and of nonparametric statistics in CASP9. Here, we have adopted the same ranking procedure as last time, but we have also confirmed that using mean and standard deviation to compute the *Z*_Q_ scores and to combine them has very limited effects on the evaluation results of the top ranked groups. We arrived at the same conclusion when we considered the options to lessen the penalization of more original and risk-taking approaches, which are more likely to attain poorer performance than other predictors.

In line with previous assessments, our scoring scheme puts heavy emphasis on correct backbone atom positions, because improvements in more fine-grained features of model quality can hardly be useful without the former. However, we observe that 049 (FEIG), 473 (Seok), 453 (KnowMin), and 222 (MULTICOM-CONSTRUCT) would still be top ranked, had we equally weighted the five evaluation measures. Only 049 (FEIG) remains consistently at the top of the ranked list, even when we leave each evaluation measure out of the calculations. Team 473 (Seok) drops from second rank only when GDT-HA is removed from the scoring scheme.

**Figure 4:**
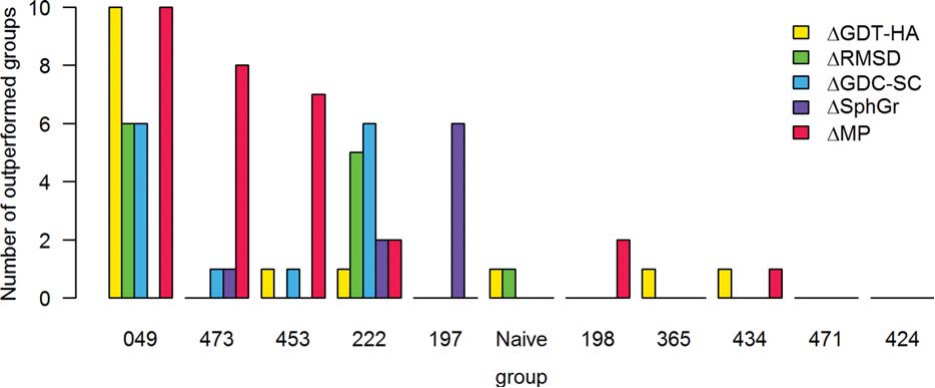
Pairwise statistical comparisons among the top 11 groups. For each group and evaluation measure, the bars represent the number of outperformed groups (significance level of 0.01) on the set of common targets.

The results presented so far do not help us understand whether the participants had concentrated on specific aspects of refinement in their prediction work. To this end, we carried out a more in-depth analysis of the top 11 predictors' performance via head-to-head statistical comparisons, similar to the original CASP7 TBM assessor's proposal.^32^ Here, we look at areas where individual groups tend to outperform the others—see Figure 4—rather than simply using the outcome of such hypothesis tests for ranking purposes.

The most striking finding is that 049 (FEIG) significantly outstrips all other groups when GDT-HA improvements are considered; in terms of Δ_RMSD_, most pairs of predictors appear to be statistically indistinguishable, because the underlying measurements tend to be dominated by remaining errors rather than rewarding the improved regions. Noticeably, the best groups exhibit an exceptional ability to fix the starting models' local stereochemistry—a lesson that they have clearly learnt since CASP9. Finally, 197 (Mufold) obtains Δ_SphGr_ values significantly better than other teams included in this study; however, we would like to add a word of caution, because at this stage it is not clear yet what is the impact of residue solvent accessibility and secondary structure on *SphGr* scores.

Finally, we monitored the impact of residues characterized as having high flexibility (i.e., high temperature factors) or forming crystal contacts as judged by visual inspection of the available experimental structural data using PyMOL's symmetry generation option. After discarding the flagged residues from the evaluation, we were only able to carry out this last test on GDT-HA scores, because we could only compute the Δ_Q_ values for measures that were based solely on alpha carbon atoms. Here, too, the overall assessment results changed only to a limited degree—see Supporting Information Table S2. Finally, we checked to see if the assessment measures correlated at all with the resolution or R-factor of the target structures, but saw no evidence for this (data not shown), suggesting that the underlying quality of the experimental data itself was not likely to affect the ranking in any significant way.

**Figure 5:**
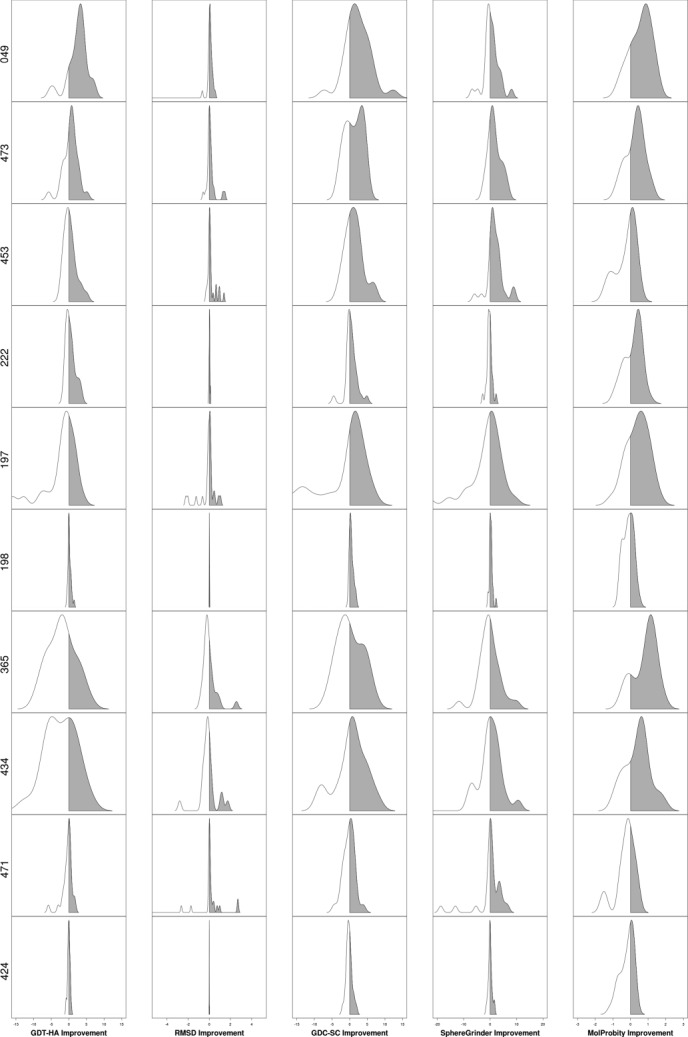
Kernel density plots showing aggregate results for the top 10 groups for all targets, using cherry-picked models. Groups are ordered by their overall performance considering their “Model 1” for each target.

Figure 5 confirms that different groups perform better at different aspects of refinement, while there is some variation in consistency across all metrics. Groups 049 (FEIG) and 473 (Seok) are the most consistent as they improve on (or equal) the starting model across all metrics more often than not, and both groups improve GDT-HA in a clear majority of cases. Groups 477 (BAKER) and 149 (wfFUIK) improved GDT-HA significantly less frequently but their best models improve GDT-HA by the largest amount, with the same being true of MP scores. 049 (FEIG), 197 (Mufold), and149 (wfFUIK) are the top groups at side chain positioning, with 049 (FEIG) again making the largest improvements, while 197 (Mufold) and 473 (Seok) are best at improving SphGr scores.

### Protein model refinement and ranking ability

**Figure 6:**
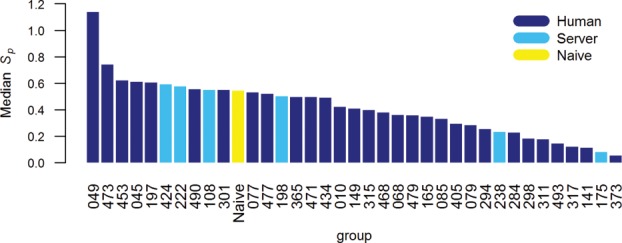
Median *S*_p_ scores based on the best models that each group submitted for each target. Only data for predictors trying to refine at least 23 targets are plotted.

A long established aspect of the CASP experiment is that up to five models for the same target can be submitted by each participating group, with the caveat that the evaluation will place main emphasis on the primary model. The data corresponding to additional models can assist predictors in testing slightly different versions of their methods or in exploring completely different strategies—but this is something virtually impossible for assessors to deal with in a sensible way. It is clearly unfair to construct a ranking of groups where single submitted models from one group are compared to models cherry-picked from a set of five from another group. Nevertheless, we were interested in knowing whether any of the supplementary predictions would in fact offer better solutions to the refinement problems. For this purpose, we first selected the best (cherry-picked) prediction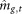 that each group *g* had produced for a specific target *t*. This was done by pooling together all assessed submissions for *t*, then calculating the associated *S*_p_ scores using this larger population of models, and finally taking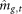 as the entry from *g* with largest *S*_p_ score. We took the median of the target-based *S*_p_ value distributions for those teams predicting 23 or more targets, and we plotted them in Figure 6. The underlying statistics for all assessed groups can be found in the Supporting Information Table S3. Overall, as might be expected, an increased number of participants attain scores higher than the baseline method when cherry-picking is allowed, but the top-ranked groups remain essentially the same.

**Figure 7:**
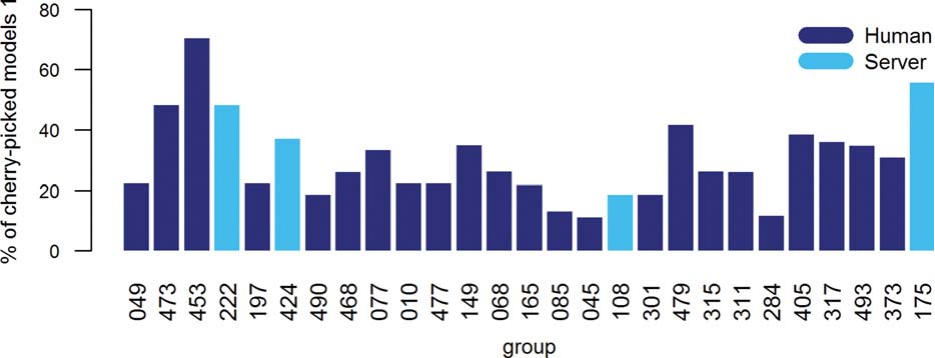
Percentage of best entries correctly labeled as model 1 that each team submitted. Bars are shown only for predictors providing 5 models for 23 or more targets. Groups are listed according to the official ranking based on model 1 data.

Because there is no formal requirement for predictors to rank their five models (other than the reasonable expectation that model 1 should be the best), it is not possible to go too far in evaluating the ability of groups to correctly identify the best model from a set of decoys. However, out of curiosity we did look to see to what extent predictors had correctly labeled their best model as model 1. To examine this, we used the same data set as described above, and for each group we calculated the percentage of predicted targets for which model 1 would have been cherry-picked *a posteriori*. A graphical summary of the results of this analysis for groups submitting five models for 23 or more targets is included in Figure 7. The results of this simple analysis show that there is no correlation between the predictors' ability to identify the best decoys and their refinement capability. At first sight this may look disappointing, but it is important to remember that predictors were not required to explicitly rank their models. These results are most likely simply down to the different “gaming” strategies being used, or in some cases evidence that predictors were indeed interested in trying quite different approaches for their own research interest. If we were to try to make any sense of the data, we might take a closer look at the level of variation in all model quality evaluation measures across all targets, and suggest that some groups—such as 473 (Seok), 453 (KnowMin), and 222 (MULTICOM-CONSTRUCT)—tend to make rather conservative changes to the starting models, and these changes may be easier to rank. Contrary to this, group 049 (FEIG) generally makes more substantial alterations to the starting models, which clearly increase the chance of making a significant move closer to the native conformation, but at the expense of giving a lower confidence level in which of these bolder moves is best. This aspect of the CASP refinement experiment certainly calls for a lot more attention in the future.

### Molecular replacement

One important aspect of refinement is to ask whether the improvements being made have any practical benefit. We already know that the refined models are still closer to the starting models than they are to the experimental structure, but are there applications where even the current fairly conservative levels of refinement are still useful? Along similar lines to previous studies, we assessed the feasibility of using these models for X-ray molecular replacement (MR) in the 13 cases where the target structure was solved by X-ray crystallography and the necessary structure factor data were available before the final meeting. Table3 shows the LLG scores for starting models and the best model for each of the 13 targets for which suitable experimental was available. Results indicate that in all cases, the best refined models produce a higher LLG score than the starting model, while in three cases, refinement results in LLG scores >60 where the starting model scores were <60, suggesting that the structure of these proteins may have been solvable in a completely automated way only when using the best refined model. These results clearly suggest that refinement can be of practical benefit to X-ray crystallographers. Additionally, the relative fast compute time required to scan through a large number of models in search of a good solution means that the difficulty in identifying the best models need not impede the use of refinement techniques for MR. In terms of group performance, we also examined how frequently individual groups were able to produce models with LLG >60 (Table4). Ten groups were able to produce at least one model above this threshold, though the top six groups are distinct in succeeding in producing models with LLG >60 in between 8 and 11 of the 13 cases.

**Table III:** Molecular Replacement Results Showing LLG Scores for Starting Models and Best Predictions

Target	Starting model's LLG	Best prediction	Best model's LLG
TR644	146.95	TR644TS124_1	275.13
TR663	43.31	TR663TS085_2	80.10
TR671	47.29	TR671TS479_4	60.23
TR674	96.42	TR674TS028_1	174.63
TR679	29.68	TR679TS197_2	54.35
TR681	392.24	TR681TS473_3	891.81
TR688	162.88	TR688TS049_2	193.26
TR689	914.95	TR689TS108_4	1097.58
TR704	62.07	TR704TS284_4	103.99
TR705	62.06	TR705TS473_1	68.55
TR712	910.16	TR712TS049_3	1127.80
TR747	51.40	TR747TS165_2	71.51
TR752	116.31	TR752TS197_4	181.24

**Table IV:** Number of Targets Where MR LLG Scores Were >60, Indicating a Viable MR Solution Would likely be Found

Group Id	Predictions with LLG > 60	Average ΔLLG
197	11	24.63
049	10	49.76
108	10	16.41
301	10	16.41
222	9	10.38
473	8	29.22

### Refinement assessment ideas for future CASPs

After we had completed the core assessment and were happy that we had come up with a robust overall ranking of groups, we spent a little time exploring other ideas that might be polished up for use in future refinement sections of CASP.

### Evaluating hydrogen bonding

Although the GDC-SC, SphGr, and MP scores aim at gauging relatively finer aspects of model quality than GDT-HA and RMSD typically do, most of these metrics suffer from being fairly hard to interpret by nonspecialists. RMSD tends to be well understood by theoreticians and experimentalists alike, but the other scores generally do not give experimentalists any intuitive feel as to how they relate to physical (or chemical) reality. In CASP7, the TBM assessor suggested that examining the correctness of H-bond assignments could help make the evaluation more detailed and intuitive.^32^ This step was taken further in CASP8 with the distinction between the interactions involving only main chain atoms and those established by all other pairs of atoms.^25^,^33^ One positive aspect of this view of model quality is that experimental structural biologists are much more familiar with aspects of hydrogen bond geometry than they are with GDT-HA scores, for example. There are clearly many technical difficulties in considering hydrogen bonding for model quality evaluation. Firstly, how hydrogen bonds should be objectively defined from a set of protein coordinates. Secondly, the relationship between a protein fold and its hydrogen bonding is variable. For example, a beta-sheet rich structure will clearly be more sensitive to hydrogen bond network disruption than a mainly helical structure. In this pilot study, we felt that the issue of protein fold bias was probably less of an issue. However, we acknowledge that some kind of weighting depending on the mixture of short-range versus long-range hydrogen bonding would be necessary in a full-fledged investigation.

We computed precision and recall for both main chain and side chain atom pairs by using the HBPLUS program^29^ to define hydrogen bonds in both models and experimental structures. The hydrogen bonds found in the experimental structures were treated as the observations (binary variables) and the hydrogen bonds found in the models treated as the predictions. A model that has the exact same set of hydrogen bonds as the experimental structure would end up with a precision of 1.0 and recall of 1.0, and therefore an F_1_ score also of 1.0. main chain hydrogen bond results turned out to be well correlated with the other measures we had used in assessment; therefore groups attaining high *S*_p_ scores tended to rank similarly in this test as well (data not shown). To our surprise, however, the side chain results appeared entirely anomalous; with many more local side chain H-bonds in many of the models than were observed in the experimental structures. After the CASP meeting, it became clear that this issue mainly affects predictions obtained from MD simulations as compared to those from other approaches—for example, template or fragment-based methods. It is likely that such inconsistencies arise from surface residues, which would otherwise be competing with the surrounding solvent for H-bonding potential. Possibly the differences are simply down to the fact that the experimental structures are time averaged and the MD models are snapshots taken at a particular time point. Certainly, factoring residue solvent accessibility in the analysis might help rationalize the results and establish a more comprehensive and meaningful picture in the future. Finally, we would like to stress that both predicted and experimental structures lack explicit coordinates for H atoms. HBPLUS attempts to position hydrogen atoms in a reasonably unbiased, yet admittedly crude way, so these findings should be treated with caution.

### Evaluating energetic changes using FlexE

One thing that we felt was slightly lacking from our assessment was finding a sensible way to properly reward more adventurous approaches. Of course the backbone metrics do give some idea as to which groups made the largest overall move towards the native structure, but this tells only part of the story. Some quite large changes in RMSD, for example, can be easily achieved in some cases by making fairly simple adjustments to a model. A good example of this would be rigid body domain motions, where just a few main chain torsion angle adjustments can radically change the relative orientation of a pair of domains and greatly reduce the calculated RMSD. Another aspect might be the relative difficulty between making changes near the termini of a model compared to carrying out radical changes to the core strands in a buried sheet.

Our suggestion here is that future refinement assessments might take into account some kind of analysis of the network of interresidue distances in the native structure. Making improvements to highly connected parts of a model should in some way be given more credit than simply reorienting rigid bodies sharing few contacts. Having used FlexE to measure deformation energy during target selection, we were keen to see if it could be used for this purpose too. This would entail using FlexE in a nonstandard way, as normally the reference structure for FlexE is assumed to be the experimental structure. In our application, we asked FlexE to calculate the energy changes between the starting model, as reference, and each of the submitted models. Therefore, a way of rewarding more adventurous groups would be to assign higher weight to models that produce the higher energy changes to the starting models. This proved rather harder to get right than we first thought, since larger FlexE scores (e.g., >5 kcal/mol/residue) resulted in models with negative ΔGDT-HA in a clear majority (∼88%) of cases; a future strategy may therefore be to scale the overall score for a given target by the FlexE score only when ΔGDT-HA is positive. Where ΔGDT-HA was positive, FlexE scores were only >5 kcal/mol/residue in ∼6% of cases for top ranked models; in two cases models with energy differences >25 kcal/mol/residue were produced, albeit with a maximum ΔGDT-HA of 3.79, while the FlexE energy difference of the model with the highest ΔGDT-HA value of 7.03 was only 4.46 kcal/mol/residue. Averaging FlexE scores across all top ranked models, groups 477 (BAKER) and 149 (wfFUIK) appeared to be the most adventurous in their submitted models, with relatively large energy changes although this came at the expense of improving GDT-HA in only 4 or 5 cases (Table5). The next best group was 473 (Seok), who struck a fairly good balance between adventurous and conservative strategies.

**Table V:** Average and Maximum FlexE Energy Scores (kcal/mol/residue) for the Top 10 Ranked Groups Where “Model 1” ΔGDT-HA Was Positive

Group Id	Targets with ΔGDT-HA > 0	Average FlexE	Max. FlexE
049	24	0.31	0.91
473	16	1.15	10.34
197	9	0.91	3.22
453	15	0.29	0.99
077	7	0.51	1.40
222	12	0.15	0.34
477	4	5.51	11.79
149	5	2.60	7.32
424	8	0.33	1.28
365	11	0.06	0.09

Although these results are interesting, we still feel that further work is needed to get this aspect of future assessments properly integrated. Getting the right balance between conservative and adventurous strategies is just as much a problem for assessors as it is for the predictors.

## DISCUSSION

### Progress since CASP9

At every CASP meeting, the same important question is always asked: has there been progress since the last CASP? Progress since CASP9 is hard to measure for a variety of the usual reasons: targets vary, and in the absence of an objective measure of target difficulty it is impossible to determine how comparable CASP9 and CASP10 targets are. Target difficulty has always proven hard to quantify, even for the simpler category of TBM, and for model refinement we really have no idea how it might be done. Even ignoring the issues of target difficulty, direct comparison with the tabulated results from the previous assessor is complicated by the fact that we have also modified the overall ranking score slightly since CASP9, with negative *Z*_Q_ scores now being set to zero. Despite this, we can look at the results qualitatively, and observe that group 049 (FEIG) has made consistent improvements across the clear majority of targets, and with almost double the number of targets since CASP9, the significance of this achievement alone must be very high. Comparing the average overall scores for the groups that participated in both CASP9 and CASP10, Group 049 (FEIG) is the only group showing a significant improvement (Δ*S*_p_ = 0.75), with 473 (Seok) and 453 (KnowMIN) showing little or no change, and 477 (BAKER) apparently performing slightly worse. The results for group 453 were certainly consistent with the expectations of the method's developers, as they confirmed the method itself was essentially unchanged from CASP9. Another observation we can make is that, for the first time, the best refinement strategy was based on molecular dynamic simulations, with previous CASP top-ranked refinement teams having relied upon knowledge-based statistical potentials. It would be inappropriate to assign a positive or negative direction to this change, as there is no reason to consider MD to be inherently better (or worse) than knowledge-based approaches. However, it certainly adds to the variety of successful methods available, and at the very least it suggests that the promise of MD in the field of protein modeling, having disappointed for so long, may at last be bearing fruit.

### Ideas for future CASP refinement experiments

Our experiences in assessing the CASP10 refinement category have led us to the following suggestions for future experiments:

– The addition of a scoring term to reward adventurous groups that make higher energy changes to structures should be considered. This would serve as encouragement for the community to attempt larger remodeling and to push predictions closer to the target structure than the starting model.
– The use of MP scores is perhaps a little incongruous with other aspects of the evaluation procedure, as it is essentially a statistical evaluation method rather than a method based around direct comparison with an experimental structure. A further issue is that groups may be over-fitting their algorithms to satisfy the narrow requirements of Molprobity, and that a broader range of methods aimed at assessing stereochemical compatibility between models and the experimental structures may be needed to properly ensure progress in this area.
– Assessing improvement since the previous CASP is very difficult, given that refinement algorithms, targets and groups have changed. Asking groups to submit predictions using a freeze of their method from the previous CASP, in addition to predictions using their most recent methods, would allow assessors to research whether a particular group had made progress. Even if a small number of groups participated in this way, it would provide a more objective measure of progress as a community than it is currently available.
– Some groups were clearly following a strategy of selecting alternative starting models from server models rather than actually trying to refine the given starting model as intended. This strategy was generally unsuccessful, mainly because we had already picked the starting models from the best available server models. Because CASP does not lay down rules as to which method should be used, these predictors cannot be blamed for deciding to follow that strategy. Clearly, however, it is not in the spirit of the experiment that we are trying to carry out. At the very least this may require some kind of clarification to predictors in the future, though at the end of the day it is almost impossible to police. The only complete solution would probably be for predictors to make their code available for independent testing.
– There is some doubt as to whether the provision of hints is useful, partly as it seems that the majority of groups do not use them. There is also the question of how realistic it is to be told which parts of a model might need special attention.
– Despite good progress in selecting additional targets compared to the past, more targets are always going to allow deeper analysis. We feel that this time around we had sufficient targets to do the basic job of assessment, but still not enough targets to address more detailed questions such as what parts of models are being modeled best by different approaches.

## CONCLUSIONS

Our assessment of the CASP10 refinement category suggests that there has indeed been important progress since CASP9 several groups achieved better results than the naïve control method, while the top ranked group (FEIG) has demonstrated high consistency in successfully improving almost all of the starting models. The strategy of this predictor is relatively conservative in terms of conformational changes, although other groups such as 477 (BAKER) were more adventurous and achieved some success albeit at the expense of consistency. In a few sporadic cases, the various Foldit groups, who employed large distributed networks of humans playing the Foldit game for model refinement, produced the best submitted model for a target. Unfortunately, they submitted models further away from native than the starting model more frequently. Most groups also demonstrated a clear improvement in their ability to produce stereochemically sound models, even when they were not able to improve the overall accuracy of the starting model. Being able to improve stereochemical quality substantially, without significantly reducing starting model accuracy, is a useful result when taken in isolation.

Despite these very satisfying improvements compared to CASP9, a number of challenges clearly remain. While five groups outperform the naïve method, the majority of groups do not-that is they effectively degrade the starting model more often than they improve it. The absolute minimum requirement for a refinement method is to at least “do no harm” to a given model. Also, it must be said that successful refinement efforts generally result in fairly modest improvements, with no groups managing to produce models that are more similar to the native state than to the starting model. The latter issue suggests that much broader sampling strategies will be needed if momentum in this category is to be maintained. And much like CASP9, there is still no evidence that groups are able to pick up the best model from the alternatives generated. In fairness, the experiment is not set up to properly evaluate decoy selection for refined models, and not every predictor even submits alternative models, so we cannot make definitive statements on this point. However, the fact that there were significantly better models submitted that were not entered as the primary model seems to suggest that the problem of properly scoring models in the endgame of protein modeling still remains unsolved.

No matter what benchmarking strategy is taken, ultimately the true test of an algorithm or methodology is whether or not it has any practical value in the real world. Refined models may be measurably and statistically better than the starting models, but are they better in any truly useful way? We should therefore like to finish on a positive note by pointing out that, in general, the top groups were indeed able to produce refined models that are more useful than the starting models in an important real-world application, namely that of X-ray molecular replacement. These results are extremely encouraging and will hopefully help drive the community to greater successes in the model refinement field.
