# Supplementary material for: Evaluation of predictions in the CASP10 model refinement category
Source: Proteins. 2014 Jul 31;82(Suppl 2):98–111. doi: 10.1002/prot.24377 (PMC4282348; doi:10.1002/prot.24377)
Supplement: Supplementary file 1 [file prot0082-0098-SD1.docx]

**Table S1.** Summary statistics of the raw scores attained by each assessed group.

| **Group** | **n** | **median** *S_p_* | **median**  **Δ*_GDT-HA_*** | **median Δ***_RMSD_* | **median**  **Δ***_GDC-SC_* | **median Δ***_SphGr_* | **median Δ***_MP_* |
| --- | --- | --- | --- | --- | --- | --- | --- |
| 049 | 27 | 0.992 | 2.530 | -0.045 | 1.310 | 0.000 | -0.810 |
| 441 | 1 | 0.864 | -13.260 | -0.710 | -7.000 | 0.760 | -1.370 |
| 124 | 2 | 0.812 | 0.200 | -0.296 | 4.525 | 1.060 | -0.730 |
| 473 | 27 | 0.674 | 0.570 | -0.011 | 1.210 | 1.030 | -0.480 |
| 292 | 2 | 0.623 | -0.970 | -0.201 | 2.410 | -0.100 | -0.725 |
| 453 | 27 | 0.506 | 0.210 | -0.014 | 0.480 | 0.000 | -0.420 |
| 222 | 27 | 0.486 | 0.000 | -0.007 | 0.460 | 0.000 | 0.070 |
| 028 | 18 | 0.484 | -1.955 | 0.051 | 0.755 | -3.325 | -1.765 |
| 197 | 27 | 0.460 | -0.420 | -0.030 | 0.400 | 1.610 | 0.200 |
| Naive | 27 | 0.450 | 0.000 | 0.000 | 0.000 | 0.000 | 0.000 |
| 198 | 26 | 0.444 | 0.000 | 0.004 | -0.215 | 0.000 | 0.090 |
| 365 | 27 | 0.409 | 0.000 | 0.003 | -0.450 | 0.000 | 0.080 |
| 434 | 27 | 0.409 | 0.000 | 0.004 | -0.450 | 0.000 | 0.050 |
| 471 | 27 | 0.407 | 0.000 | 0.003 | -0.310 | 0.000 | 0.100 |
| 424 | 27 | 0.388 | -0.530 | -0.003 | -0.670 | 0.000 | 0.230 |
| 490 | 27 | 0.384 | -0.840 | 0.022 | -0.620 | 0.520 | -0.210 |
| 103 | 2 | 0.362 | -3.940 | 0.097 | 0.560 | 1.685 | -0.490 |
| 468 | 27 | 0.303 | -5.320 | 0.215 | -1.410 | -0.550 | -1.210 |
| 077 | 27 | 0.274 | -2.940 | 0.093 | 0.610 | -0.760 | -0.500 |
| 010 | 27 | 0.271 | -1.700 | 0.031 | -1.730 | -1.550 | -0.130 |
| 477 | 27 | 0.264 | -4.920 | 0.240 | -3.370 | -2.030 | -1.330 |
| 341 | 20 | 0.246 | -6.230 | 0.418 | -2.145 | -2.280 | -1.225 |
| 149 | 23 | 0.245 | -6.080 | 0.273 | -0.970 | -1.110 | -0.630 |
| 068 | 23 | 0.237 | -8.820 | 0.679 | -3.400 | -2.860 | -1.310 |
| 165 | 23 | 0.223 | -9.120 | 0.432 | -3.210 | -3.660 | -1.270 |
| 085 | 23 | 0.221 | -9.140 | 0.611 | -4.410 | -4.200 | -1.250 |
| 045 | 27 | 0.220 | -1.330 | 0.025 | -0.120 | 1.200 | -0.190 |
| 260 | 17 | 0.210 | -5.250 | 0.113 | -4.330 | 0.000 | 1.000 |
| 108 | 27 | 0.205 | -1.510 | 0.207 | -0.510 | -0.420 | 0.170 |
| 301 | 27 | 0.205 | -1.510 | 0.207 | -0.510 | -0.420 | 0.170 |
| 079 | 27 | 0.201 | -1.340 | 0.000 | -2.200 | -1.620 | 0.770 |
| 479 | 26 | 0.179 | -1.990 | 0.160 | -3.820 | -3.535 | 0.575 |
| 315 | 24 | 0.168 | -3.490 | 0.039 | -2.380 | -0.825 | 0.960 |
| 311 | 27 | 0.147 | -7.980 | 0.488 | -4.900 | -3.300 | -0.510 |
| 284 | 26 | 0.140 | -5.760 | 0.243 | -3.110 | -3.625 | -0.825 |
| 405 | 27 | 0.127 | -2.680 | 0.142 | -3.240 | 0.000 | 0.660 |
| 406 | 7 | 0.122 | -10.200 | 1.333 | -4.260 | -5.100 | -0.610 |
| 286 | 1 | 0.118 | -8.150 | 2.160 | -0.730 | -0.710 | -1.180 |
| 294 | 27 | 0.091 | -0.990 | 0.043 | -1.510 | -1.000 | 0.440 |
| 238 | 26 | 0.088 | -0.960 | 0.043 | -1.625 | -1.070 | 0.435 |
| 317 | 27 | 0.087 | -5.260 | 0.099 | -5.660 | -3.250 | 0.150 |
| 298 | 23 | 0.066 | -2.290 | 0.082 | -1.890 | -1.110 | 0.190 |
| 493 | 26 | 0.061 | -3.035 | 0.223 | -1.630 | -4.310 | 0.415 |
| 373 | 27 | 0.052 | -11.150 | 0.505 | -9.520 | -14.040 | -0.390 |
| 141 | 27 | 0.042 | -3.700 | 0.276 | -1.700 | -0.570 | 0.800 |
| 175 | 27 | 0.020 | -5.110 | 0.210 | -2.850 | -2.220 | 1.220 |
| 006 | 21 | 0.000 | -6.250 | 0.757 | -8.350 | -9.920 | 1.000 |
| 201 | 5 | 0.000 | -16.490 | 1.741 | -7.740 | -15.130 | 1.130 |

For each group, the table reports the number of model 1 predictions officially assessed (n), the median of the *S_p_* scores, as well as the median of the differences in model quality measures relative to the starting models. Data sorted by descending median *S_p_* values.

**Table S2.** Effect of alternative assessment choices on group ranking.

| **Official ranking** | **Using parametric statistics** | **No clipping of *Z_Q_*** | **Clipping of negative *S_p_*** | **Evenly weighted Z*_Q_*** | **No Δ*_GDT-HA_*** | **No Δ***_RMSD_* | **No Δ*_GDC-SC_*** | **No Δ*_SphGr_*** | **No Δ*_MP_*** | **No uncertain amino acids** |
| --- | --- | --- | --- | --- | --- | --- | --- | --- | --- | --- |
| 049 | 049 | 049 | 049 | 049 | 049 | 049 | 049 | 049 | 049 | 049 |
| 473 | 473 | 473 | 473 | 473 | 468 | 473 | 473 | 473 | 473 | 453 |
| 453 | 453 | 453 | 453 | 453 | 473 | 453 | 453 | 453 | 453 | 197 |
| 222 | 222 | 222 | 222 | 222 | 477 | 222 | 197 | 222 | 197 | 473 |
| 197 | Naive | 198 | 198 | 197 | 068 | Naive | Naive | Naive | 222 | 222 |
| Naive | 197 | Naïve | Naive | Naïve | 165 | 471 | 222 | 197 | Naïve | 385 |
| 198 | 365 | 365 | 365 | 198 | 085 | 198 | 424 | 198 | 198 | 471 |
| 365 | 198 | 434 | 434 | 365 | 197 | 365 | 365 | 434 | 434 | 434 |
| 434 | 434 | 471 | 471 | 434 | 453 | 434 | 198 | 365 | 424 | 365 |

The table reports changes in relative group performance across a range of evaluation options.

**Table S3.** Median *Sp* scores attained by the participating groups based on their best submissions for each target.

| **Group** | **Type** | **n** | **median *S_p_*** |
| --- | --- | --- | --- |
| 049 | H | 27 | 1.14 |
| 292 | S | 2 | 1.10 |
| 124 | S | 2 | 1.01 |
| 473 | H | 27 | 0.74 |
| 453 | H | 27 | 0.62 |
| 045 | H | 27 | 0.61 |
| 197 | H | 27 | 0.61 |
| 424 | S | 27 | 0.59 |
| 222 | S | 27 | 0.58 |
| 490 | H | 27 | 0.55 |
| 108 | S | 27 | 0.55 |
| 301 | H | 27 | 0.55 |
| 103 | S | 2 | 0.54 |
| Naive | NA | 27 | 0.54 |
| 077 | H | 27 | 0.53 |
| 477 | H | 27 | 0.52 |
| 198 | S | 26 | 0.50 |
| 365 | H | 27 | 0.50 |
| 471 | H | 27 | 0.50 |
| 434 | H | 27 | 0.49 |
| 028 | S | 18 | 0.44 |
| 010 | H | 27 | 0.42 |
| 149 | H | 23 | 0.41 |
| 341 | H | 20 | 0.40 |
| 315 | H | 24 | 0.40 |
| 260 | H | 17 | 0.38 |
| 468 | H | 27 | 0.38 |
| 068 | H | 23 | 0.36 |
| 479 | H | 26 | 0.36 |
| 165 | H | 23 | 0.35 |
| 085 | H | 23 | 0.33 |
| 405 | H | 27 | 0.29 |
| 079 | H | 27 | 0.28 |
| 441 | H | 1 | 0.28 |
| 294 | H | 27 | 0.25 |
| 238 | S | 26 | 0.23 |
| 284 | H | 26 | 0.23 |
| 298 | H | 23 | 0.18 |
| 311 | H | 27 | 0.18 |
| 286 | S | 1 | 0.15 |
| 493 | H | 26 | 0.15 |
| 406 | H | 7 | 0.14 |
| 317 | H | 27 | 0.12 |
| 141 | H | 27 | 0.11 |
| 175 | S | 27 | 0.08 |
| 373 | H | 27 | 0.05 |
| 006 | S | 22 | 0.01 |
| 201 | H | 5 | 0.00 |

For each group, the table reports whether the group registered as human (H) or server (S), the number of predicted targets (n), and the median *S_p_* score assigned to the cherry-picked models. Data sorted by descending median *S_p_* scores. NA: Not Available.
